# Three-dimensional ankle kinematics in children’s school shoes during running

**DOI:** 10.1186/1757-1146-5-S1-O20

**Published:** 2012-04-10

**Authors:** Caleb Wegener, Damien O’Meara, Adrienne E Hunt, Joshua Burns, Benedicte Vanwanseele, Andrew Greene, Richard M Smith

**Affiliations:** 1 Discipline of Exercise and Sports Science, Faculty of Health Sciences, The University of Sydney, NSW, 1825, Australia; 2 New South Wales Institute of Sport, Sydney, NSW, 2129, Australia; 3 Faculty of Health Sciences, The University of Sydney / Institute for Neuroscience and Muscle Research, The Children’s Hospital at Westmead, Sydney, NSW, 2145, Australia; 4 Research Centre for Exercise and Health, KULeuven, Leuven, Belgium / Chair Health Innovation and Technology, Fontys University of Applied Sciences, Eindhoven, Netherlands

## Background

Children are more active during the school day than at other times [1] and because school shoes are required as part of a uniform in many countries research on school shoes is required. This study aimed to determine the effect of school shoes on the ankle joint complex motion of children while running.

## Materials and methods

Twenty children (mean age 9 years (SD2.3)) performed five running trials at a self-selected velocity barefoot and wearing school shoes (Daytona, Clarks) in a random order. A 14 camera 200Hz motion analysis system (EVaRT5.0, MAC) was used to calculate marker trajectories. Markers were attached to the right leg and a cluster wand was attached to the calcaneus through a window in the shoe. A standing reference trial was used to embed segment axes and then calculate ankle joint complex motion. Force plate data were collected at 1000Hz (Kistler™). Data were normalised to the stance phase and sub-phases partitioned from the anterior/posterior force data as: loading (initial-contact – maximum-negative force); mid-stance (maximum-negative force – zero) and propulsion (positive force – toe-off).

## Results

Shoes delayed the maximum-posterior force (22.8% to 29.3%; *p*<0.0001) and the zero crossing of the anterior-posterior force (41.1% to 43.6%; *p*=0.021). During loading shoes increased ankle range of motion (ROM) in the sagittal (9.9° to 13.8°; *p*=0.007) and transverse planes (5.7° to 7.7°; *p*=0.007). During midstance shoes decreased ankle frontal plane ROM (3.7° to 2.8°; *p*=0.037). During propulsion shoes increased ankle ROM in the sagittal plan (30.3° to 33.3°; *p*=0.018) and decreased frontal plane ROM (14.4° to 12.0°; *p*=0.042). Overall stance phase sagittal plane ROM increased in shoes (31.2° to 34.2°; *p*=0.034).

## Conclusions

This study shows that school shoes increase sagittal ankle motion during loading and propulsion, but decrease frontal plane motion during mid-stance and propulsion. These findings will assist in harmonising school shoe design with foot function.
